# Web-based intervention to promote weight-loss maintenance using an activity monitor: A randomized controlled trial^☆^

**DOI:** 10.1016/j.pmedr.2019.100839

**Published:** 2019-03-04

**Authors:** Yoshio Nakata, Hiroyuki Sasai, Takehiko Tsujimoto, Koichi Hashimoto, Hiroyuki Kobayashi

**Affiliations:** a Faculty of Health and Sport Sciences, University of Tsukuba, Tsukuba, Japan; b Graduate School of Arts and Sciences, The University of Tokyo, Japan; c Faculty of Human Sciences, Shimane University, Japan; d Faculty of Medicine, University of Tsukuba, Tsukuba, Japan

**Keywords:** BMI, body mass index, CI, confidence interval, FG, food group, HDL, high-density lipoprotein, ITT, intention-to-treat, MET, metabolic equivalents, MVPA, moderate-to-vigorous physical activity, RCT, randomized controlled trial, SD, SansDisk, UMIN, University Hospital Medical Information Network, USB, Universal Serial Bus, Body weight changes, Exercise, Diet, Obesity management

## Abstract

The present study examined whether a web-based intervention could promote weight-loss maintenance, after weight loss. The study was a two-phase, 27-month, randomized controlled trial conducted in Ibaraki, Japan, from 2014 to 2017; 133 participants were recruited through local newspaper advertisements. The eligibility criteria were as follows: age of 40–64 years, body mass index of 25–40 kg/m^2^, and having at least one metabolic syndrome component. In phase 1, a 3-month, group-based weight-loss program was provided to all eligible participants (*n* = 119). We then randomly assigned (1:1) participants who had lost 5% or more of their weight during phase 1 (*n* = 95) to either the self-help (mean 3-month weight loss 7.30 kg) or the web-support group (7.00 kg). Participants in the web-support group regularly reported their body weight and physical activity through a web-based system. They received monthly personalized feedback from a study staff for 24 months. The primary outcome, 27-month body-weight change (mean ± standard deviation), in the self-help and web-support groups were − 5.3 ± 5.0 kg and −4.5 ± 4.9 kg, respectively. There was no significant difference. An exploratory secondary analysis demonstrated that those with greater 27-month increases in their step count, assessed with an accelerometer, lost more weight with no difference in changes in energy intake. The mean 27-month body-weight change in the 4th quartile of changes in step count was −7.78 kg. Although web-based intervention using an activity monitor failed to promote weight-loss maintenance, increased physical activity was associated with successful weight-loss maintenance.

## Highlights

• Web-based intervention using an activity monitor delivered no benefit on weight.
• Participants with larger increases in step count lost more weight.
• Increased physical activity was associated with successful weight loss maintenance.

## 1 Introduction

Obesity is one of the most salient public health problems in developed and developing countries (Finucane et al., 2011). A recent systematic review and meta-analysis demonstrated that weight-loss intervention for obese adults might reduce premature death (Ma et al., 2017), which supports public health efforts to prevent weight gain and facilitate weight loss. Diet (Johns et al., 2014) and structured exercise training (Umpierre et al., 2011) were shown as key components of weight management program. Web-based intervention programs were also shown as effective strategies to promote and maintain weight loss (Hutchesson et al., 2015; Sherrington et al., 2016; Sorgente et al., 2017). However, most previous studies were conducted in the United States and European countries.

Despite the fact that Japan currently enjoys the world's highest life expectancy, obesity epidemic remains a problem that has not been fully controlled. The mean body mass index (BMI) increased from 22.1 kg/m^2^ to 23.5 kg/m^2^ in men and from 21.3 kg/m^2^ to 23.3 kg/m^2^ in women between 1980 and 2008 (Finucane et al., 2011). The Japanese Ministry of Health, Labour and Welfare's current strategy, developed in response to the scourge of obesity (Tsushita et al., 2018), needs to be revised and improved. Our research team has conducted several intervention studies to encourage weight loss in overweight and obese Japanese adults, developing an effective weight-loss program, with 6–8 kg lost in 3–6 months (Nakata et al., 2009, Nakata et al., 2011). The program consisted of a motivational lecture, educational materials such as textbooks and diaries, and group-based support sessions. Each component was shown to be effective in a 6-month randomized controlled trial (RCT) (Nakata et al., 2011). Furthermore, 24-month, intervention-free follow-up revealed that the effectiveness of a 6-month, group-based intervention disappeared at the end of the 24-month follow-up, and that a weight-loss maintenance program was necessary (Nakata et al., 2014).

Several observational studies have suggested that increased physical activity is associated with successful weight-loss maintenance. For example, participants in the National Weight Control Registry, which is the prospective investigation of long-term successful weight-loss maintenance, were extremely physically active (Catenacci et al., 2008). A prospective cohort study showed that increased physical activity, particularly high-intensity physical activity, was associated with better weight-loss maintenance (Mekary et al., 2010). In our previous study (Nakata et al., 2014), in a secondary analysis, participants were divided into quartile groups based on percentage weight loss. The results showed that the highest quartile group (i.e., successful weight-loss maintainers) had significantly greater step counts (+2607 steps/day) and moderate-to-vigorous physical activity (MVPA) (+21 min/day) than the lowest quartile group (−33 steps/day and −4 min/day). These results suggest that increasing physical activity contributes to successful weight-loss maintenance.

In order to increase and maintain physical activity levels, web-based intervention seemed an attractive option; it would save time and labor, and may have a modest effect on weight control (Kodama et al., 2012). Given that the web-based intervention performs adequately well, the weight loss could be maintained after the 24-month follow-up, in contrast to the finding of our previous study (Nakata et al., 2014). Thus, the purpose of the present study was to test whether web-based intervention could be used to maintain weight loss by encouraging physical activity.

## 2 Methods

### 2.1 Design and participants

The present study was registered at the University Hospital Medical Information Network (UMIN) Clinical Trials Registry (UMIN000014428) and comprised 3 months of weight loss (phase 1) followed by 24 months of weight-loss maintenance (phase 2). Participants were recruited through newspaper advertisements in two cities (Mito and Chikusei) in Ibaraki Prefecture, Japan, from August to November 2014. Community centers in these cities were used for this study. The eligibility criteria were as follows: 40–64 years of age, BMI of 25–40 kg/m^2^, and at least one component of metabolic syndrome (abdominal obesity, hypertension, dyslipidemia, or hyperglycemia), based on the Japanese criteria (Tsushita et al., 2018). Ineligibility criteria included a history of coronary disease or stroke, current or planned pregnancy, participation in another weight-loss program during the previous 6 months, and participation of cohabitants in this study. At the time of enrollment into the present study, we confirm that participants had personal computers and access to the internet.

During phase 1, all participants underwent our verified weight-loss program (Nakata et al., 2009, Nakata et al., 2011), which involved changes in diet and exercise. Specifically, the recommendations included an energy-restricted diet of 1200 and 1600 kcal/day for women and men, respectively; as well as a minimum physical activity increase of 1000 kcal/week. We provided textbooks, notebooks and group-based support sessions during weeks 1, 2, 3, 4, 6, 8, 10, and 12. The content of the textbook and notebook was based on the Four-Food-Group Point Method as shown in our previous study (Nakata et al., 2011). In this method, all foods were grouped into four food groups (FG) based on the nutrient contents: FG 1 (eggs and dairy products), FG 2 (protein-rich products such as meat, fish, and soybean), FG 3 (vitamin-rich products such as vegetables and fruits), and FG 4 (energy-rich products such as carbohydrates and oil). To calculate energy intakes and to balance the nutrients easily, an 80-kcal food translated into 1 point. The 1200 and 1600 kcal/day for women and men corresponded to 15 and 20 points, respectively. Both men and women consumed 3-point foods from FGs 1–3 (9 points in total), and the remaining points were given to FG 4. In this phase, during the final lecture, participants were instructed on how to adjust the dietary strategy after phase 1 in order to maintain their weight loss, including the need to carefully increase the energy intake mainly from FG 4, by 1 or 2 points per day, by keeping the consumption from FGs 1–3.

Participants who lost 5% or more of their baseline body weight were eligible for the subsequent 24-month weight-loss maintenance phase. They were then randomly assigned (1:1) into the self-help or web-support groups, which were stratified by city and gender using simple computerized randomization procedures. An investigator, who had no contact with the participants or other staff members, generated the group allocation data, which were retained at a secure central location until the eligible participants were determined. Assuming a difference of 2.5 (4.0) kg in the 27-month weight change between groups, an alpha level of 5%, and a power of 80%, the required sample size for the final analysis was 84 in total. Assuming an eligibility rate of 90%, an achievement rate of 90% for 5% loss of baseline weight, and a drop-out rate of 20% in phase 2, the required sample size assessed for eligibility was determined to be 130. The Institutional Review Board of the University of Tsukuba approved the protocol. Participants were informed about the aim and design of the study, including random assignment and the importance of minimizing the dropout rate to maintain the study data quality; and their informed consent was obtained. Data collection and the study intervention were conducted until February 2017.

### 2.2 Weight-loss maintenance interventions

**Fig. 1:**
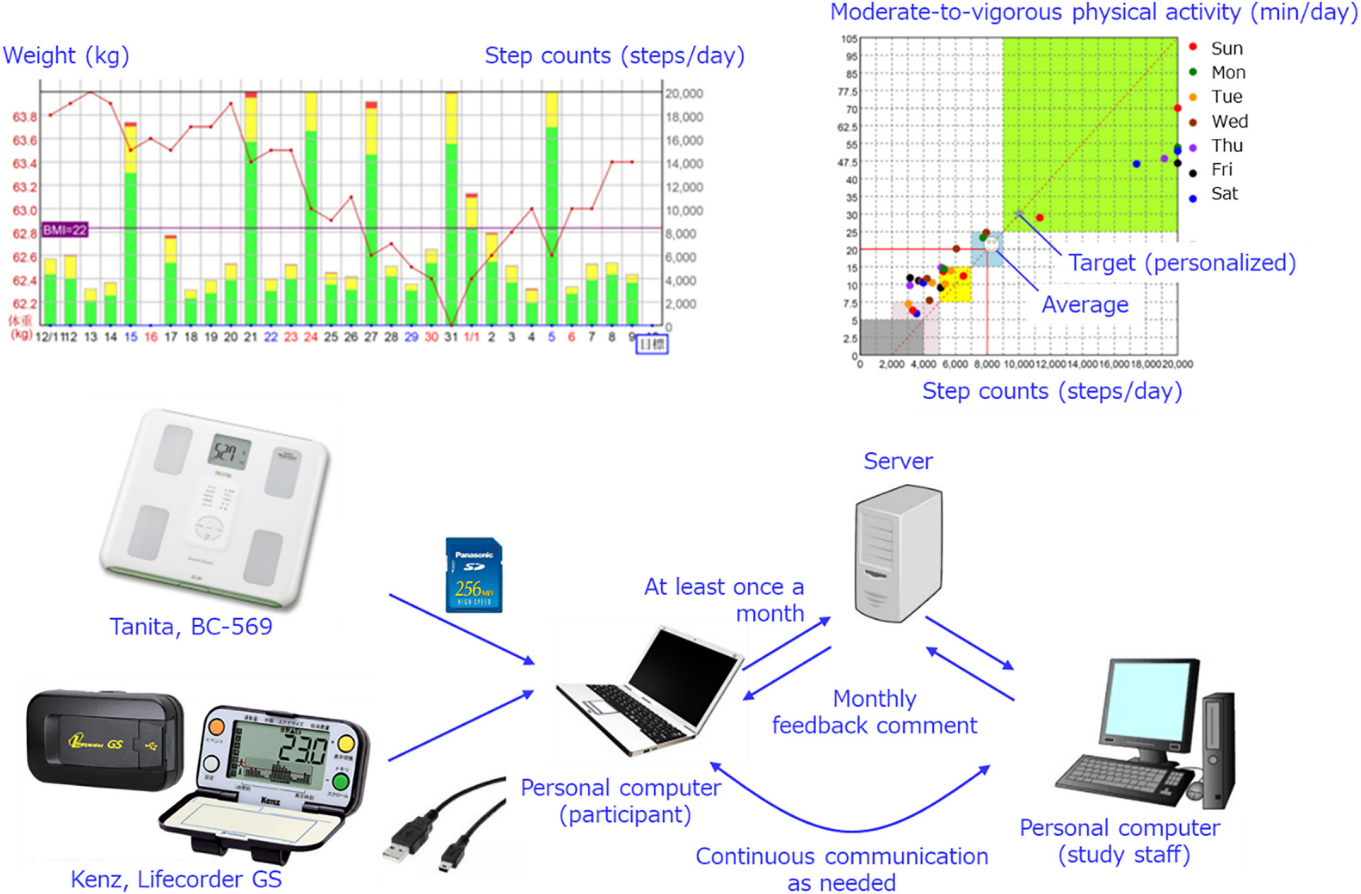
Web-based system using weight scale and activity monitor (Ibaraki, Japan, 2014–2017).

The self-help group was not provided with any additional intervention during phase 2; instead, they made self-help efforts toward weight-loss maintenance. The web-support group was provided with a weight scale (BC-569; Tanita, Tokyo, Japan) and an activity monitor (Kenz Lifecorder GS; Suzuken, Nagoya, Japan), and received intervention via a web-based, weight-loss maintenance program (Fig. 1). Participants in the web-support group were instructed to measure their body weight daily and to wear the activity monitor at the waist during waking hours. With the use of their personal computers, they regularly (at least once a month) logged into the web-based system using their original ID and passwords; and uploaded their body weight and physical activity data. Thus, their personal computers had to be compatible with the SansDisk (SD) card inserted in the weight scale, as well as with the activity monitor, which used a Universal Serial Bus (USB) cable. The web-based system automatically created two graphs of the participants' activity patterns, one showing changes in body weight and step count (upper left in Fig. 1) and another displaying a scatter diagram consisting of step count (X axis) and MVPA (Y axis; upper right in Fig. 1) (Aoyagi and Shephard, 2013). The default step count and MVPA targets per day were set at 8000 steps and 20 min, respectively; however, the personal targets were set individually with a planned gradual increase (500–2000 steps/day and 5–20 min/day). One author (Y.N.) checked the participants' body weight and physical activity using the web-based system and provided monthly personalized feedback (approximately 350–450 Japanese characters, corresponding to 200–250 words in English) for 24 months. Typical advices comprised of monthly evaluation of weight and physical activity, applause and encouragement. Although dietary record was not included in the web-based system, with increasing body weight and increasing physical activity, some concerns about diet were included in the feedback messages. The participants could view the feedback messages when they logged into the web-based system on their personal computers or mobile phones. We could not obtain the frequency of logging in, or whether the participants read the messages. If the body weight or physical activity data were not uploaded, the study staff contacted the participant via e-mail or by phone, once a month, to ask that the data be uploaded. Other communication (via the web-based system, e-mail or by phone) with the study staff was allowed as needed.

### 2.3 Measurements

The measurement visits were held at the baseline (month 0), the end of phase 1 (month 3), the middle (month 15), and the end of phase 2 (month 27). The primary outcome was the 27-month change in body weight, while the secondary outcomes were 27-month changes in waist circumference, systolic and diastolic blood pressure, triglycerides, high-density lipoprotein (HDL) cholesterol, and fasting plasma glucose. As compliance measures, total energy intake and physical activity were also assessed. All study outcomes were measured by trained staff members, as described below.

#### 2.3.1 Demographics, lifestyle, and medication

Using a self-administered questionnaire delivered at baseline, participants reported their age, sex, smoking status, and prescribed medication for hypertension, dyslipidemia, and diabetes.

#### 2.3.2 Anthropometrics

Height was measured to the nearest 0.1 cm using a portable stadiometer (213; Seca, Hamburg, Germany), and body weight was measured to the nearest 0.05 kg using a calibrated digital scale (WB-150; Tanita, Tokyo, Japan). The participants' BMI was computed as their weights in kg divided by the square of their heights in meters. Waist circumference was measured twice to the nearest 0.1 cm at the umbilicus level using a flexible plastic tape, with the participant in the standing position, as described in the standard guidelines of the Japanese nationwide health check-up program (Tsushita et al., 2018). The value of the two measurements was averaged and used for data analysis.

#### 2.3.3 Blood pressure

To measure blood pressure, the participants were seated for ≥5 min with their arm supported at heart level. Systolic and diastolic blood pressure was then ascertained on the arm using an automated sphygmomanometer (HEM-7430; Omron Healthcare, Kyoto, Japan). The average value of the two measurements was used for data analysis.

#### 2.3.4 Blood biochemistry

Venous blood was collected from each participant after an overnight fast of ≥12 h. Serum triglyceride levels were determined enzymatically (Determiner L TG II; Kyowa Medex, Tokyo, Japan). Serum HDL cholesterol was measured using the selective inhibition method (MetaboLead HDL-C; Kyowa Medex). Blood glucose was assayed using the hexokinase-G-6-phosphate dehydrogenase method (L-Type Glu 2; Wako Pure Chemical Industries, Osaka, Japan). The blood samples were assayed by an independent laboratory (Kotobiken Medical Laboratories, Ibaraki, Japan).

#### 2.3.5 Dietary intake

Total energy intake, in kilocalories, was quantified using 3-day weighed food records, which was used as a reference for the dietary intake (Sasaki et al., 1998). The participants were instructed to record every food item they ate for 3 days, including 2 weekdays and 1 weekend day. They measured the food using standard measuring cups, spoons, and digital scales. To assure overall comparability, a skilled nutritionist, who was blinded to the group allocations, reviewed and analyzed all food records using a computer program (Eiyoukun, Kenpakusya, version 6.0, Tokyo, Japan) with the Japanese food composition tables (2010 edition).

#### 2.3.6 Physical activity

The participants were instructed to wear a tri-axial accelerometer (Active style Pro HJA-350IT; Omron Healthcare) at the waist for 14 consecutive days. The participants' daily steps and intensity of physical activity (expressed as metabolic equivalents [METs]) were counted and estimated by the accelerometer using a validated algorithm (Ohkawara et al., 2011; Oshima et al., 2010). The participants did not wear the devices while sleeping, while engaged in a water-based activity (e.g., bathing or swimming), or while participating in activities such as contact sports (for safety reasons). A valid day was defined as a wear time of ≥10 h (Troiano et al., 2008). If no acceleration signal, over a 1-minute time interval, was obtained for 60 consecutive minutes or more, the period was defined as “non-wear” (Mâsse et al., 2005). When valid data were obtained for ≥3 days, the daily step count and time spent in MVPA (≥3 METs) were summarized for each participant (Mâsse et al., 2005).

### 2.4 Statistical analysis

All data handling and statistical analyses were conducted in full accordance with the analysis plan in the study protocol, using the open-source statistical environment R (3.2.4 for Windows 64-bit). All *p*-values <0.05 were deemed statistically significant. The participants' baseline characteristics were shown as mean ± standard deviation for continuous variables, or as frequency and percentage for categorical variables. Our primary analysis followed an intention-to-treat (ITT) principle, with missing data imputed by baseline observations carried forward. Changes in 27-month primary, secondary, and compliance outcomes are described as means with 95% confidence intervals (CIs). The unpaired Student's *t*-test was used to examine the statistical significance of changes in 27-month between-group differences. Furthermore, a graph of weight trajectories by group allocation was created using means and standard errors. For the exploratory secondary analysis, the participants were retrospectively categorized into quartiles of 27-month change in step count. Differences among the quartiles were examined using one-way analysis of variance for continuous variables and the *χ*^2^ test for categorical variables.

## 3 Results

**Fig. 2:**
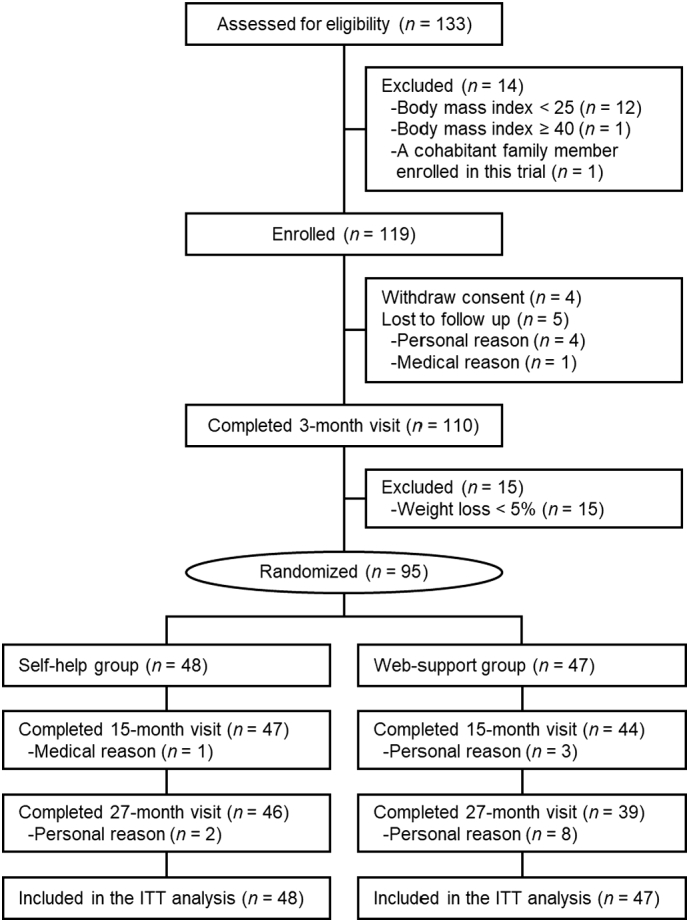
Participant flowchart: intention-to-treat (ITT) (Ibaraki, Japan, 2014–2017).

The study flowchart is presented in Fig. 2. A total of 133 candidates were assessed for eligibility, and 119 met the eligibility criteria. The eligible participants were then enrolled in the initial 3-month weight-loss phase. Of these, 110 (92.4%) completed the 3-month intervention, and 95 (79.8%) lost ≥5% of their body weight. Ultimately, the 95 participants were randomized into the self-help group (*n* = 48) and the web-support group (*n* = 47) and served in the primary ITT analyses. As noted in Table 1, there were no notable differences in baseline characteristics between the groups.

**Table 1:** Baseline characteristics of intervention participants (*n* = 95) (Ibaraki, Japan, 2014–2017).

Characteristic	Self-help group (*n* = 48)	Web-support group (*n* = 47)
Demographics and lifestyle		
Age, years	57.0 (5.7)	54.7 (6.6)
Female gender, *n* (%)	30 (62.5)	29 (61.7)
Current smoking, *n* (%)	3 (6.2)	2 (4.3)
Medication use		
Antihypertensive, *n* (%)	17 (35.4)	17 (36.2)
Lipid-lowering, *n* (%)	14 (29.2)	11 (23.4)
Hypoglycemic, *n* (%)	1 (2.1)	1 (2.1)
Anthropometrics		
Height, cm	162.2 (8.1)	162.2 (8.3)
Weight, kg	74.2 (8.1)	74.7 (10.6)
3-month weight change, kg	−7.30 (−8.03, −6.57)	−7.00 (−7.62, −6.38)
Body mass index, kg/m^2^	28.2 (2.4)	28.4 (3.1)
Waist circumference, cm	98.6 (6.3)	98.8 (7.4)
Blood pressure		
Systolic, mm Hg	138.7 (14.6)	136.8 (16.5)
Diastolic, mm Hg	87.8 (9.6)	84.5 (10.5)
Blood biochemistry		
Triglycerides, mg/dL	132.3 (92.9)	127.3 (103.9)
HDL cholesterol, mg/dL	58.3 (15.9)	57.2 (15.3)
Fasting plasma glucose, mg/dL	102.3 (26.3)	97.3 (11.4)
Energy intake and physical activity		
Total energy intake, kcal/day	1993 (407)	2039 (398)
Valid days, day	13.6 (1.8)	13.6 (1.5)
Wear time, min/day	929.9 (73.6)	943.0 (85.7)
Step count, steps/day	6167 (2409)	6246 (2689)
MVPA, min/day	57.2 (25.2)	57.5 (30.3)

Notes: Data are expressed as mean (standard deviation) for baseline values and as mean (95% confidence interval) for 3-month changes, unless otherwise specified.

Abbreviations: HDL, high-density lipoprotein; MVPA, moderate-to-vigorous physical activity.

Of those who took part in phase 2, 46 (95.8%) in the self-help group and 39 (83.0%) in the web-support group completed the 27-month visit. The retention rate in the self-help group was significantly higher than in the web-support group (*p* = 0.04). The missed assessments were mostly due to personal or medical reasons unrelated to the study. In the web-support group, the participants uploaded their body weight on a median (first–third quartile) of 75.6% (43.1%–83.4%) of days during the 24-month weight-maintenance phase. More specifically, in months 4–9, 10–15, 16–21, and 22–27, the equivalent proportions were 80.2% (53.9%–92.6%), 81.0% (48.8%–91.0%), 61.5% (31.8%–83.3%), and 71.4% (12.8%–84.2%), respectively. Similarly, the participants in the web-support group uploaded their step count on a median of 69.4% (55.5%–79.8%) of days during the 24-month weight-loss maintenance phase. The equivalent proportions were 68.1% (58.2%–78.9%), 70.7% (61.3%–85.6%), 77.5% (58.5%–86.6%), and 69.4% (29.8%–81.4%) in months 4–9, 10–15, 16–21, and 22–27, respectively. During phase 2, some technical issues were raised about the activity monitor (*n* = 14), network (*n* = 8), personal computer (*n* = 6), SD card (*n* = 5), USB cable (*n* = 2), and weight scale (*n* = 2). Consultation and/or exchange of devices were made as necessary. No clinically significant adverse events occurred that were judged by the investigators to be related to participation in the trial.

**Fig. 3:**
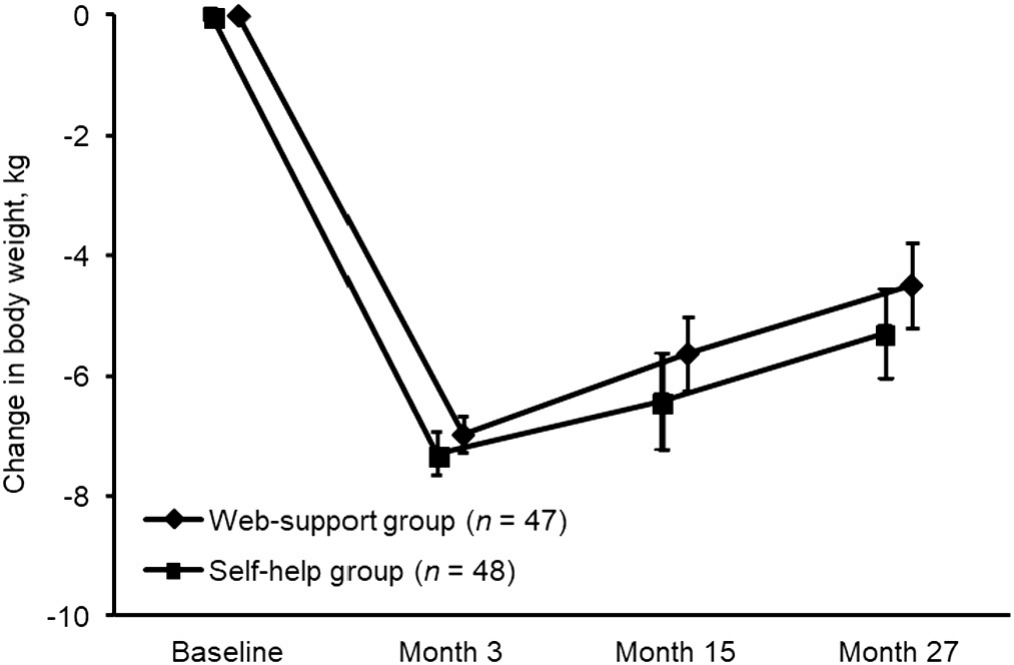
Pattern of change in body weight by group assignment. Each data point indicates the mean value of all randomized participants, with missing values imputed by baseline observations carried forward. Error bars indicate standard errors (Ibaraki, Japan, 2014–2017).

Table 2 shows the ITT analyses of the primary, secondary and compliance outcomes. Apart from those who missed the 27-month visit, four participants showed invalid physical activity data at month 27: one in the self-help group and three in the web-support group. The data from the four participants were treated as missing, and then replaced by the participants' baseline values. Although the 95%CI of changes in almost all outcomes showed significant improvements in each group during the 27-month period, none of the improvements significantly differed between the two groups. Fig. 3 depicts the time course of body weight among all randomized participants. There were no significant differences at any time point between the two groups.

**Table 2:** Changes in body weight and metabolic syndrome components over 27 months (*n* = 95) (Ibaraki, Japan, 2014–2017).

Characteristic	Self-help group (*n* = 48)	Web-support group (*n* = 47)	*p*-value
Anthropometrics			
Weight, kg	−5.30 (−6.76, −3.84)	−4.50 (−5.93, −3.07)	0.43
Weight loss ≥5%, *n* (%)	28 (58.3)	26 (55.3)	0.93
Body mass index, kg/m^2^	−1.97 (−2.51, −1.44)	−1.74 (−2.28, −1.19)	0.53
Waist circumference, cm	−5.40 (−6.96, −3.84)	−4.54 (−5.85, −3.22)	0.40
Blood pressure			
Systolic, mm Hg	−0.22 (−4.66, 4.22)	0.49 (−3.37, 4.35)	0.81
Diastolic, mm Hg	−2.30 (−5.06, 0.45)	−1.09 (−3.31, 1.14)	0.49
Blood biochemistry			
Triglycerides, mg/dL	−29.2 (−47.5, −10.9)	−18.8 (−34.3, −3.3)	0.38
HDL cholesterol, mg/dL	4.44 (2.17, 6.70)	4.30 (1.53, 7.06)	0.94
Fasting plasma glucose, mg/dL	−4.98 (−9.22, −0.74)	−0.40 (−3.07, 2.26)	0.07
Energy intake and physical activity			
Total energy intake, kcal/day	−264.9 (−374.6, −155.2)	−314.4 (−433.5, −195.3)	0.54
Step count, steps/day	33.5 (−485.7, 552.7)	532.5 (−71.5, 1136.5)	0.21
MVPA, min/day	−0.34 (−6.46, 5.77)	3.62 (−3.09, 10.34)	0.38

Notes: Data expressed as mean (95% confidence interval) unless specified. Invalid data on physical activity (*n* = 4) were replaced by their baseline values.

Abbreviations: HDL, high-density lipoprotein; MVPA, moderate-to-vigorous physical activity.

Table 3 shows the baseline characteristics and changes in outcome measures among retrospectively defined quartiles of 27-month change in step count based on the data of those who completed the study with no missing data (*n* = 81). Irrespective of either of the randomized groups (i.e., self-help or web-support), those who increased their step count lost more weight and attained a weight loss of ≥5% at a higher rate during the 27-month study period with no difference in 27-month changes in energy intake.

**Table 3:** Baseline characteristics and 27-month changes in outcome measures according to the quartiles of change in step count (*n* = 81) (Ibaraki, Japan, 2014–2017).

Characteristic	27-month change in step count				*p*-value
	1st quartile (≤−860 steps, *n* = 21)	2nd quartile (−860–325 steps, *n* = 20)	3rd quartile (325–1626 steps, *n* = 20)	4th quartile (≥1626 steps, *n* = 20)	
Age, years	56.5 (5.4)	56.0 (6.0)	57.0 (6.2)	55.2 (7.4)	0.82
Female, *n* (%)	13 (61.9)	13 (65.0)	12 (60.0)	12 (60.0)	0.99
Web-support group, *n* (%)	8 (38.1)	7 (35.0)	10 (50.0)	11 (55.0)	0.53
Current smoking, *n* (%)	1 (4.8)	1 (5.0)	2 (10.0)	0 (0.0)	0.55
Antihypertensive medication, *n* (%)	10 (47.6)	7 (35.0)	5 (25.0)	7 (35.0)	0.51
Lipid-lowering medication, *n* (%)	9 (42.9)	2 (10.0)	4 (20.0)	8 (40.0)	0.06
Hypoglycemic medication, *n* (%)	1 (4.8)	1 (5.0)	0 (0.0)	0 (0.0)	0.57
Height, cm	161.8 (8.9)	160.2 (8.6)	163.4 (7.0)	162.6 (8.4)	0.65
Weight, kg					
Baseline	70.9 (7.6)	73.3 (10.1)	75.6 (10.6)	76.0 (9.1)	0.28
Change	−4.34 (−6.02, −2.67)	−4.02 (−5.97, −2.06)	−6.88 (−9.51, −4.25)	−7.78 (−9.48, −6.08)	0.02
Loss of ≥5%, n (%)	11 (52.4)	10 (50.0)	13 (65.0)	18 (90.0)	0.03
Body mass index, kg/m^2^					
Baseline	27.1 (1.7)	28.5 (3.1)	28.2 (2.8)	28.7 (2.4)	0.16
Change	−1.65 (−2.26, −1.03)	−1.49 (−2.20, −0.79)	−2.55 (−3.52, −1.59)	−3.00 (−3.71, −2.28)	0.01
Waist circumference, cm					
Baseline	96.7 (5.7)	98.2 (7.1)	99.3 (6.3)	98.6 (4.3)	0.53
Change	−4.39 (−6.25, −2.53)	−4.94 (−7.41, −2.46)	−6.76 (−9.14, −4.38)	−7.11 (−9.17, −5.05)	0.19
Systolic blood pressure, mm Hg					
Baseline	138.0 (16.6)	142.1 (17.4)	139.8 (12.8)	134.9 (11.7)	0.48
Change	0.90 (−7.33, 9.14)	−1.42 (−6.83, 3.98)	−0.40 (−5.50, 4.70)	1.90 (−4.69, 8.49)	0.88
Diastolic blood pressure, mm Hg					
Baseline	87.0 (9.8)	87.4 (10.4)	84.5 (7.5)	87.5 (9.3)	0.73
Change	−0.19 (−5.09, 4.71)	−2.92 (−7.18, 1.33)	−2.60 (−5.40, 0.20)	−2.62 (−6.35, 1.10)	0.72
Triglycerides, mg/dL					
Baseline	115.3 (47.8)	137.1 (143.0)	128.0 (119.6)	157.9 (81.3)	0.61
Change	−24.6 (−41.2, −8.0)	−14.9 (−42.2, 12.3)	−29.5 (−61.9, 2.9)	−48.0 (−81.5, −14.4)	0.37
HDL cholesterol, mg/dL					
Baseline	60.6 (15.6)	55.8 (13.9)	59.4 (17.6)	52.4 (11.4)	0.29
Change	4.67 (0.07, 9.26)	4.20 (1.20, 7.20)	4.65 (1.02, 8.28)	7.45 (2.81, 12.09)	0.63
Fasting plasma glucose, mg/dL					
Baseline	100.3 (37.2)	104.0 (17.3)	97.5 (10.0)	99.7 (11.7)	0.83
Change	−6.43 (−14.65, 1.80)	0.90 (−4.09, 5.89)	−3.15 (−7.69, 1.39)	−4.20 (−9.66, 1.26)	0.34
Total energy intake, kcal/day					
Baseline	1975 (359)	2130 (513)	1977 (479)	1988 (332)	0.60
Change	−281.5 (−468.3, −94.6)	−429.9 (−663.2, −196.7)	−308.5 (−462.4, −154.7)	−257.7 (−412.3, −103.0)	0.53
Step count, steps/day					
Baseline	8065 (2426)	5560 (2234)	5612 (1758)	5920 (3105)	0.003
Change	−2202 (−2765, −1640)	−289 (−472, −105)	928 (742, 1115)	3005 (2519, 3490)	<0.001
MVPA, min/day					
Baseline	76.2 (22.5)	52.7 (25.7)	51.2 (25.6)	55.4 (29.3)	0.008
Change	−20.9 (−29.6, −12.3)	0.2 (−7.2, 7.6)	0.6 (−5.4, 6.5)	28.9 (21.7, 36.2)	<0.001

Notes: Data expressed as mean (standard deviation) for baseline values and as mean (95% confidence interval) for 27-month changes, unless otherwise specified.

Abbreviations: HDL, high-density lipoprotein; MVPA, moderate-to-vigorous physical activity.

## 4 Discussion

The present randomized controlled trial was conducted to test whether a web-based weight-maintenance intervention that encouraged physical activity was effective at maintaining weight loss. However, the primary analysis showed no such benefit.

The web-based intervention followed the participants' body weight and physical activity. This was based on several observational studies (Catenacci et al., 2008; Mekary et al., 2010) and our previous intervention study (Nakata et al., 2014) suggesting that increased physical activity was associated with successful weight-loss maintenance. The compliance rates for weight and physical activity recording were ~75% and ~70%, respectively. The step count and MVPA targets were personally set and planned to gradually increase. Typically, the planned increase in step count was around 1500–2000 steps, while the increase in MVPA was planned as 10–15 min. However, the increase in 27-month step count and MVPA time in the web-support group were 532.5 steps and 3.62 min, respectively—slightly larger, but not significantly different from those in the self-help group. The lower-than-assumed effect on physical activity was the primary reason why web-based intervention yielded no benefit. Therefore, a new approach may be necessary to increase the step count and MVPA. In previous studies, successful approaches were suggested. Leahey et al. (2016) demonstrated the effectiveness of an internet-delivered approach on weight loss maintenance in a 10-month maintenance intervention. To increase the benefit, in the successful intervention above, social and monetary reinforcements were used. Furthermore, to mitigate boredom, different strategies were implemented, with changes in specific strategies every 2 weeks. Voils et al. (2017) reported the effectiveness of weight maintenance intervention after a 16-week weight loss on a 56-week weight regain. The maintenance intervention above commenced with group visits to initiate maintenance skills at weeks 2, 6, and 10, and individual telephone calls at weeks 4, 8, 12, 16, 20, 24, 32, and 40. This program was more intense than our approach, especially in the first 3 months, and this gradually decreased with frequency of contact, and might be better.

Three further potential reasons why web-based intervention showed no benefit were considered. Firstly, in the self-help group, weight regain occurred at a lower level than assumed. Specifically, during study planning, we assumed that the 27-month weight loss in the self-help group was about 3 kg, as demonstrated in our previous RCT (Nakata et al., 2014), which showed a 30-month weight loss of 3.3 kg with no weight-loss maintenance intervention. However, in the present study, the 27-month weight loss was 5.3 kg in the self-help group and 4.5 kg in the web-support group. In this regard, the self-help group might have motivated themselves to comply with appropriate behavior because of rivalry with the web-support group (since they were not selected for the intervention group). This is a limitation of open-label trial. Secondly, the 3-month weight loss in the self-help group was slightly larger than that in the web-support group. This chance between-group difference may have influenced the 27-month change in body weight. The 27-month weight loss in the self-help group was also slightly larger than in the web-support group, whereas the prevalence of weight loss ≥5% was almost the same in both groups (58.3% vs. 55.3% in the self-help and web-support groups, respectively). Thirdly, the web-based intervention did not include the assessment of dietary intake. Actually, with increasing body weight and increasing physical activity, some concerns about diet were included in the feedback messages. However, much intense monitoring of dietary intake might be necessary to improve weight loss maintenance (Johns et al., 2014).

An exploratory secondary analysis using the data of those who completed the study with no missing data (*n* = 81) demonstrated that those with greater increases in their step count lost more weight and attained a weight loss of ≥5% at a higher rate, with no difference in 27-month changes in energy intake. Specifically, the 27-month weight changes were −4.02 kg, −6.88 kg, and −7.78 kg, in the 2nd, 3rd, and 4th quartiles of 27-month step counts, respectively. Although the first quartile (≤−860 steps) showed a 27-month weight change of −4.34 kg, their mean step count at baseline was 8,065 steps, which was significantly higher than in the other groups. Taking this point into consideration, a dose-response relationship between physical activity and weight-loss maintenance might be suggested.

The major strength of this study was its RCT design, which focused on weight-loss maintenance. Most RCTs involving obesity have focused on how to decrease body weight during the weight-loss phase and have not involved weight-loss maintenance interventions (Sorgente et al., 2017). The present study was a carefully designed RCT comprising 3 months of weight loss and 24 months of weight-loss maintenance to test the effectiveness of web-based support after weight loss. Although no effectiveness was demonstrated, the exploratory secondary analysis revealed that increased physical activity was associated with successful weight-loss maintenance. Similar results were reported in another previous RCT. Jakicic et al. (2008) examined the 24-month weight-loss maintenance among four groups categorized on the basis of physical activity energy expenditure (1000 vs. 2000 kcal/week) and intensity (moderate vs. vigorous). The primary analysis showed no weight-loss difference among the groups, while secondary analysis using weight-change categories showed that successful weight-loss maintainers were more physically active. Therefore, increased physical activity was associated with successful weight loss maintenance; however, to increase physical activity in the long term is difficult and presents a challenge.

The present study also had some limitations. First, the number of participants lost to follow-up was larger in the web-support group than in the self-help group, partly because the web-based system involved closer communication. Second, all of the participants were Japanese, and there were more women than men. For these reasons, the generalizability of this study was limited. Third, we could not obtain data on the frequency of log-ins to the web-based system. The number of log-ins was found to be associated with less weight regain (Funk et al., 2010). Had these data being available, additional insight into how the intervention resulted in behavioral change might have been given.

## 5 Conclusion

The present 27-month RCT tested the effectiveness of a web-based weight-loss maintenance program using an activity monitor. However, the intervention failed to deliver any benefit over the self-help group. Participants with greater increases in their step count lost more weight. Increased physical activity was associated with successful weight-loss maintenance. A more intense and interesting intervention may be necessary to increase step count and MVPA.

## Conflicts of interest

None disclosed.

## Funding

This work was supported by the JSPS (Japan Society for the Promotion of Science; Chiyoda-ku, Japan) KAKENHI (grant number 25282203).

## Authors' contributions

Study concept and design: Nakata Y and Hashimoto K.

Intervention: Nakata Y and Sasai H.

Data acquisition: Nakata Y, Sasai H, Tsujimoto T, and Kobayashi H.

Statistical analysis: Nakata Y and Sasai H.

Interpretation: Nakata Y and Sasai H.

Writing of first draft: Nakata Y and Sasai H.

Study physician: Kobayashi H.

Overall supervision as principal investigator: Nakata Y.

All authors contributed toward revision of the manuscript and agreed to be accountable for all aspects of this work.
